# A Nice Day for an Infection? Weather Conditions and Social Contact Patterns Relevant to Influenza Transmission

**DOI:** 10.1371/journal.pone.0048695

**Published:** 2012-11-14

**Authors:** Lander Willem, Kim Van Kerckhove, Dennis L. Chao, Niel Hens, Philippe Beutels

**Affiliations:** 1 Center for Health Economics Research & Modeling of Infectious Diseases, Center for the Evaluation of Vaccinations, Vaccine and Infectious Disease Institute, University of Antwerp, Antwerp, Belgium; 2 Department of Mathematics and Computer Science, University of Antwerp, Antwerp, Belgium; 3 Interuniversitary Institute for Biostatistics and Statistical Bioinformatics (I-BioStat), Hasselt University, Diepenbeek, Belgium; 4 Center for Statistics and Quantitative Infectious Diseases, Vaccine and Infectious Disease Division, Fred Hutchinson Cancer Research Center, Seattle, Washington, United States of America; 5 School of Public Health and Community Medicine, The University of New South Wales, Sydney, Australia; National Institutes of Health, United States of America

## Abstract

Although there is no doubt that significant morbidity and mortality occur during annual influenza epidemics, the role of contextual circumstances, which catalyze seasonal influenza transmission, remains unclear. Weather conditions are believed to affect virus survival, efficiency of transmission and host immunity, but seasonality may also be driven by a tendency of people to congregate indoors during periods of bad weather. To test this hypothesis, we combined data from a social contact survey in Belgium with local weather data. In the absence of a previous in-depth weather impact analysis of social contact patterns, we explored the possibilities and identified pitfalls. We found general dominance of day-type (weekend, holiday, working day) over weather conditions, but nonetheless observed an increase in long duration contacts (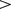
1 hour) on regular workdays with low temperatures, almost no precipitation and low absolute humidity of the air. Interestingly, these conditions are often assumed to be beneficial for virus survival and transmission. Further research is needed to establish the impact of the weather on social contacts. We recommend that future studies sample over a broad spectrum of weather conditions and day types and include a sufficiently large proportion of holiday periods and weekends.

## Introduction

Influenza is a respiratory virus that causes significant morbidity and mortality during annual epidemics and occasional pandemics [1]–[6]. Seasonal influenza is widespread in temperate climate regions during wintertime and large efforts have been made to study its seasonality. The effect of weather conditions on influenza epidemics received special attention and three main factors have been singled out: efficiency of transmission, host susceptibility and virus survival [7]–[13]. Temperature and humidity exhibit a distinct seasonality in temperate climates and absolute humidity has been hypothesized to drive influenza seasonality through modulating airborne survival and transmission [14]–[16]. A more complete understanding of viral persistence in ambient environment is required to capture the environmental effects on virus infectivity. A latitudinal shift of influenza mortality incidence was observed in Brazil from the northern, tropical regions in summer to the southern, temperate regions of the country in winter, which suggests that the virus migrates from the tropics to temperate regions in both hemispheres during winter [17]. Large respiratory particles shed by infected hosts partially evaporate when the air is dry, become smaller and are more likely to stay airborne [8]. Breathing cold, dry air reduces mucociliary clearance and phagocytic activity of the nasal passage, which normally filter pathogens from the upper respiratory tract [18]. Changes in photoperiod and sunlight exposure alter vitamin D levels and low levels have been shown to impair the body's immune response regulation [19]. Viral stability is also critical for airborne transmission and appears related to environmental factors since the lipid envelope encasing of the virus remains longer intact with cold and dry air [8], [20]. Given the sensitivity of airborne transmission to climatological factors, it is believed to be the dominant mode of transmission in temperate regions [8].

Social contact behavior is also known to be important for infectious disease dynamics [21]–[27]. Social contact patterns are prone to change given contextual changes. For instance, social mixing has been analyzed with respect to day-type (e.g., working days, weekend days and holidays) and generally lower contact rates with more intergenerational mixing are observed on weekends and holiday periods compared to working days [25], [28]. Transmission among school children tends to play a large role in influenza dynamics and both school opening and closing events have been associated with changes in influenza transmission [24], [29], [30]. Also health status interacts on social activity since an increased number of community contacts were observed during influenza seasons in adults without influenza-like illness [31]. In transmission models, mixing patterns are often parameterized based on social contact surveys, time use studies or social network analyses [21], [32]–[35], and represented in ‘Who Acquires Infection from Whom’ (WAIFW) matrices. The WAIFW matrix summarizes age-dependent transmission parameters and is a determinant of the basic reproduction number R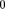, defined as the expected number of secondary infections caused by a typical primary infection in a fully susceptible population [21].

While most literature on influenza transmission aims to reveal the biological and physical mechanisms associated with different weather conditions, the influence of weather on social contact patterns remains unclear. Mikolajczyk *et al.* touched on this subject by reporting that school children reduced their contacts on rainy days by 16% [22]. Nonetheless, it is hypothesized that the seasonality of many respiratory diseases is driven in part by the tendency of people to congregate indoors when the weather is bad [7]. Analysis of human activity data showed that in addition to day-type, temperature and precipitation affect daily time usage [36]. In general, people spend 1–2 hours longer indoors during cold weather and about 0.5 hour longer during rainy days [36], [37]. However, these changes are small relative to the 21–22 hours individuals usually spend indoors. Furthermore, crowding may occur the year round at public gatherings like shopping malls, festivals, sporting events and conferences. Therefore, seasonal fluctuations in social contact patterns may not be large but could give influenza – in conjunction with other seasonal adjustments – a greater opportunity to spread during winters [7].

The aim of the current paper is to explore modified social contact patterns with respect to weather in order to give more substance to the potential relationship between climatological changes, social contact patterns and influenza seasonality. In the absence of a previous in-depth weather impact analysis of social contact patterns, we explored the possibilities and identified limitations of using existing datasets. We partitioned the social contact data according to the weather on the day of survey and estimated for each weather condition the mean number of contacts and R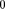 [25]. Next, relative changes were estimated by the ratio of the mean number of contacts and the R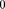 's for different weather conditions.

## Results

**Figure 1:**
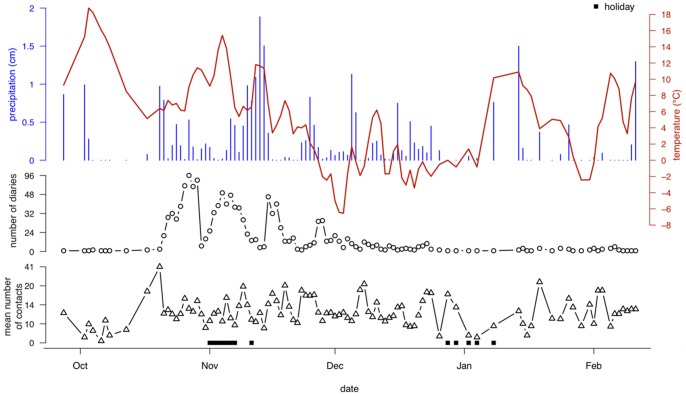
Overview of the weather and the social contact data from October 2010 until February 2011. Top: the mean daily temperature (line) and the total daily precipitation (bars). Middle: the daily number of diaries. Bottom: the mean number of social contacts per day. Holiday periods are marked at the bottom with squares.

Social contact data were obtained in Flanders between September 2010 and February 2011 and covered two school holiday periods. Figure 1 summarizes the study design by presenting the mean daily temperature, the total daily precipitation, the number of diaries and the mean number of daily contacts over time. It appears that warm weather coincided predominantly with a holiday period in early November and we observed a Spearman correlation of 0.530 between the daily temperature and holiday variables whereas the correlation with precipitation was only 0.005. Therefore, a link between daily temperatures and holiday periods had to be taken into account. Also important is the distribution of participants over time: 70% of the participants filled in their diary on 20% of the survey period (October–November).

We partitioned the dataset by the median of the mean daily temperatures (6.83

C) and calculated the ratio of the weighted mean number of contacts for each subpopulation to obtain relative changes. We used a non-parametric bootstrap to generate 95% confidence intervals (CI) and observed a moderate though non-significant increase in the number of contacts on days with low temperatures compared to days with high temperatures (mean 1.130 and CI [0.993;1.283]). The number of school contacts increased significantly (2.004 [1.363;3.027]) with cold temperatures together with contacts of at least 15 minutes (1.190 [1.039;1.341]). A stratification based on the median precipitation value (0.05 cm/day) did not yield significant results.

**Figure 2:**
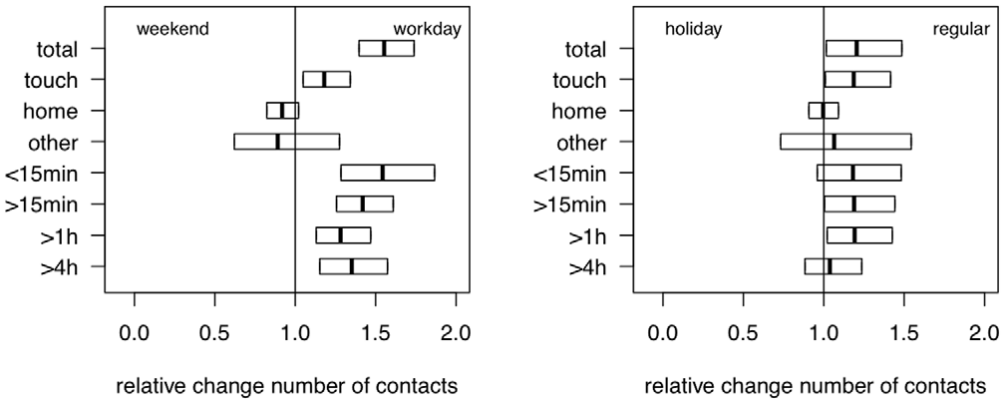
Relative change in the number of contacts for weekends (left) and holiday periods (right). The boxes present the 95% confidence interval for different contact types and durations. The relative changes are estimated by the ratio of the mean number of contact for the condition at the right side by the mean number of contact for the condition at the left side.

We validated our methods and dataset with an analysis distinguishing day-types. The boxes in Figure 2 present CI for the mean number of contacts ratio for different contact types and durations. We observed a significant increased number of contacts comparing workdays to weekends for all contacts (1.553 [1.397;1.739]) and contacts involving skin-to-skin touching (1.184 [1.049;1.342]). We found similar effects, smaller though significant, for the comparison between regular and holiday periods (Figure 2).

Given the effect of holidays on social contact behavior and the correlation between weather and holiday periods, we needed to study these periods separately to eliminate possible confounding. Stratification by working (Monday to Friday) and weekend days did not suffice to overcome the problem with relatively warm holiday periods and an initial partitioning by regular and holiday periods resulted in large uncertainty without significant results. Therefore, it was necessary to partition based on workdays and weekends during regular and holiday periods. For weekends and holiday periods, at least one age group was represented by only 3 to 5 participants, which was too limited. The age distributions of the sample populations for the temperature analysis can be found in Figure S1. The analysis for regular weekdays contains at least 17 participants in each age group hence we focused on this partition. We encountered similar problems of data sparseness for weekend and holiday periods with a stratification based on day-type and precipitation. A combined analysis with temperature and precipitation was not possible, not even for regular workdays.

We found a significant increase in the mean number of long duration contacts (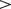
1hour) on regular weekdays with low temperatures (1.188 [1.025;1.361]) which was found to be a general tendency for all contacts longer than 15 minutes (1.143 [0.995;1.307]). The number of contacts at non-specific locations (henceforth: “non-specific contact type” during leisure, transport, family visits and other activities) decreased significantly (0.461 [0.276;0.737]) on regular workdays with low temperatures.

**Figure 3:**
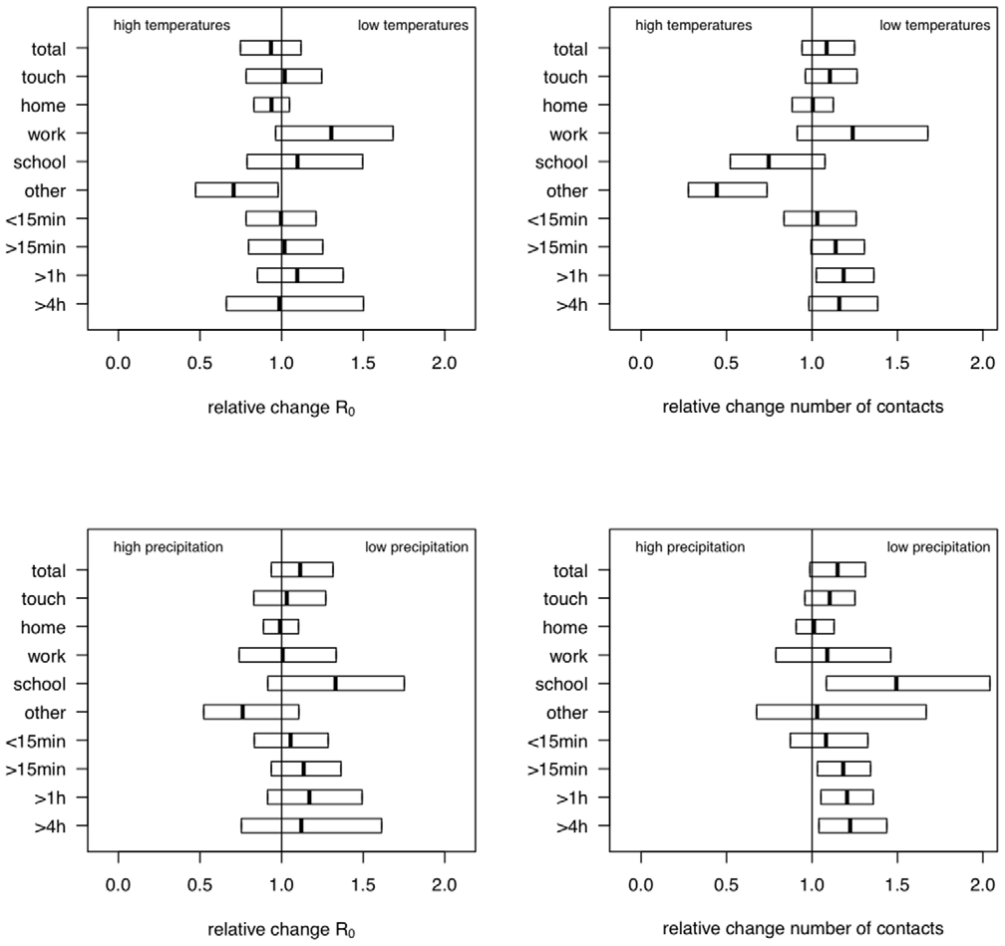
Relative change in R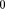 and the mean number of contacts considering daily temperatures and precipitation. The boxes present the 95% confidence interval for the R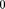 ratios (left) and the mean number of contacts (right) during regular workdays for different contact types and durations. Top: comparison between days with high and low mean daily temperature. Bottom: comparison between days with high and low precipitation.

When we analyzed social contact patterns on regular weekdays with respect to precipitation, we observed a significant increase (1.183 [1.032;1.342]) in the number of contacts longer than 15 minutes when precipitation was low (Figure 3). Also the number of school contacts showed a negative association with precipitation (1.502 [1.085;2.041]). We did not observe a change in the number of work or home contacts related to precipitation.

**Figure 4:**
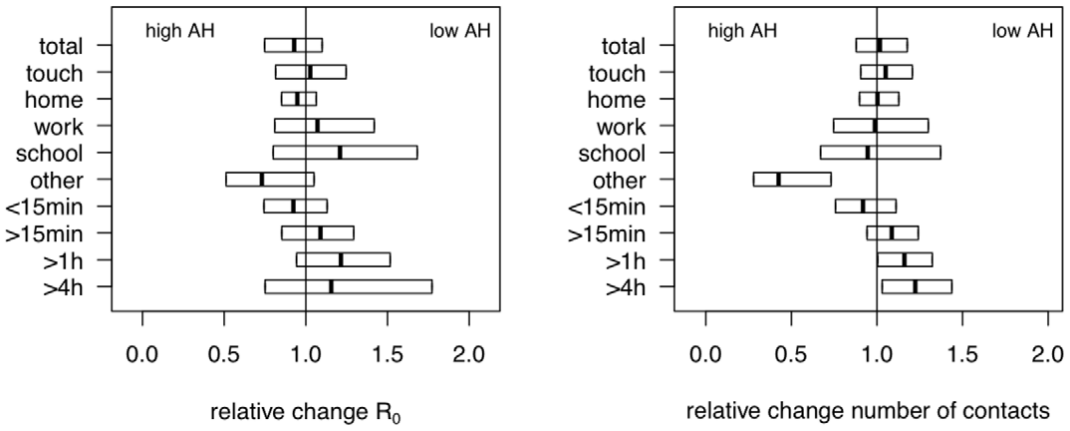
Relative change in R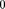 and the mean number of contacts considering absolute humidity. The boxes present the 95% confidence interval for the R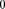 ratios (left) and the mean number of contacts (right) during regular workdays for different contact types and durations. AH: absolute humidity.

We investigated the association between absolute humidity (Figure 4) and contact patterns and observed a significant increase (1.162 [1.004;1.323]) for contacts longer than one hour with dry air if we stratified by the median absolute humidity (8.41 mbar). This atmospheric condition was also associated with a significant decrease in the number of non-specific contacts (0.445 [0.279;0.732]).

Next to contact frequency, we also focused on transmission potential by including population mixing. We calculated R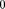 for each condition and used R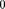 ratios to estimate relative changes. The R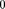 ratios for the day-types are in line with previous results, i.e. transmission decreases during weekends and holiday periods. The results for the weather conditions showed similar trends as the relative change in the number of contacts, albeit non-significant. We measured the required number of bootstrap samples to obtain stable results (Figure S2). Based on these findings, we used 1000 bootstrap samples to represent uncertainty on our results.

## Discussion

The non-weather related findings we presented were consistent with published results: people have more contacts on regular workdays compared to weekends and holidays [28]. We observed an increased number of school contacts with high temperatures and low precipitation, the latter of which is in line with a previous study in school children [22]. Additionally, the number of long duration contacts (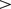
1 hour) increased on regular weekdays with low temperatures and almost no precipitation. Apart from temperature and precipitation, we observed an increased number of prolonged contacts when absolute humidity of the air was low. We also looked at transmission dynamics by calculating R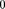 ratios for different weather conditions and observed similar trends for long duration contacts on regular weekdays with low temperature, precipitation and humidity.

The impact of weather conditions on biological mechanisms of influenza transmission has been studied, but the link with social contact patterns remains unclear [7]–[12], [16]. We presented evidence that weather may influence social mixing patterns. Although one may expect people to leave their home more often on a fine day, this does not necessarily imply that they will make more contacts. Based on the current data, we did not find a relationship between the number of contacts and the weather but the contact duration seemed to depend on the weather. One possible explanation might be that people congregate longer inside when the weather is bad, a phenomenon which has also been observed in time use studies [36], [37]. Prolonged contacts are of particular importance for infectious disease transmission since they tend to be more intensive and more often involve closer interactions [38]. Fluctuations in social contact durations together with other seasonal adjustments might give influenza a greater opportunity to spread during wintertime [10].

In order to focus on the effect of modified social contact behavior, we assumed constant transmission parameters for different weather conditions. However, temperature and humidity are likely to have some impact on virus transmission and host immunity [8], [12], [14]. Especially low absolute humidity is believed to be beneficial for influenza virus survival and transmission [14].

Proper estimation of the impact of weather conditions on social contact patterns requires considering the effect of weekend days and holiday periods. However, we experienced difficulties related to data sparseness when the data was partitioned by day-type. The dataset was designed to estimate the effect of day-types but not for an extra partitioning based on weather types. Each subpopulation has to be representative for the general population, which was not the case for weekend and holiday periods after partitioning for a weather condition. We used weights to account for different age distributions, but this was insufficient if the exposure to a particular type of weather on a particular day-type occurred for only 5 people of a single age group. The sample was only large enough on regular workdays so that our results are mainly limited to this day type. The number of unique weather conditions in our dataset was limited since the majority of the participants were recruited at the end of October and during November.

Future research in this area should carefully consider aspects of data sparseness in study design. Since weather conditions cannot be predetermined, we recommend to sample on many different days to get a broad spectrum of weather conditions. Also a sufficiently large number of respondents should participate on weekends and holiday periods to gain predictive power for these periods. Additional data on the location of the contacts – indoors or outdoors – can also lead to new insights in this challenging research field. Although we found associations between the weather and contact duration, this is no guarantee for a clear-cut causal relation with influenza seasonality. More studies are needed assessing the role of climatic conditions and contact patterns on observed epidemiological transmission patterns to provide adequate information to plan and evaluate mitigating strategies [15], [30].

## Analysis

### Social contact data

A social contact survey was conducted in the Flemish geographic region of Belgium from September 2010 until February 2011. Participants were recruited by random digit dialing on fixed and mobile telephone lines and sampling was performed in order to achieve a representative geographical spread. One person per household was recruited to take part. Sampling was undertaken to obtain an age distribution of 12.5% in the ages 0–8 and 9–24 years, 45% in 25-54 years, 20% in 55–79 years, 4% in 80–89 years and 2.4% in 90–99 years. All participants were asked to fill in a paper diary recording their contacts during one randomly assigned day without changing their usual behavior. No physical samples were collected as part of this study and the ethical committee of the Antwerp University Hospital approved the study protocol. A verbal consent was given prior to participation during the recruitment over the phone. People who agreed verbally to participate were then sent a written questionnaire and diary. The participants received and sent their questionnaires back by postal services. They were able to refuse participation even after verbal agreement by not filling in the questionnaire and diary, and/or by not sending it back. The first page of the questionnaire explained that their answers would be used anonymously for scientific research purposes at our universities. Thus, the fact that they filled in the questionnaire and diary and chose to send it in functions as a written consent. We obtained similar verbal consent with implicit written confirmation from the next of kin, caretakers or guardians on behalf of dependent participants (e.g. children).

Two types of contacts were defined: (1) a two-way conversation with a dialog of at least 3 words and (2) skin-to-skin touching either with or without conversation. Sampling days were nearly uniformly distributed between all days of the week. Information recorded in the diary included the exact or estimated age and gender of each contacted person, the duration of contacts per person over the entire day as well as the frequency (habitual nature) of contacts with that person. Furthermore, the location or circumstance of each contact was recorded. Three types of diaries were used, adapted to the ages of the participants. For example, the diaries for children (0–12 years) were designed to be filled by proxy, and included school contacts, which included separate instructions for schoolteachers, whereas those for elderly could also be filled by proxy. The diaries were sent and collected by mail. Participants were reminded by phone to fill in the diary one day in advance and followed up the day after. Data were single entered in a computer database and checked manually.

We collected and analyzed social contact data from 1752 participants. The survey was designed to sample 413 participants during a school holiday period (1–7 Nov, 11 Nov, 27 Dec –9 Jan) or during a weekend adjacent to a holiday period. Contact data were collected in this fashion of 1046 participants on regular workdays, 293 on regular weekend days, 286 on workdays during public holidays and 127 on weekend days during or adjacent to public holidays.

### Weather data

We used public data on daily precipitation and temperature from the National Oceanic and Atmospheric Administration (NOAA, http://www.noaa.gov/). There are 20 weather stations in Belgium that report mean daily temperatures (air and dew-point) based on hourly measurements. The precipitation data was of inferior quality because 631 reports were missing or inconsistent from September 2010 until February 2011. According to the NOAA documentation, some stations report “missing” on days without precipitation. Since weather conditions can vary by locality, we did not use provincial means but matched all participants to the nearest weather station with data on the day they completed the survey. We mapped the participants and the weather stations on the 19781 Belgian census tracts and used the Pythagorean theorem with the tract coordinates (Statistics Belgium, 2001) to find the nearest weather station. This way, for every participant a temperature and precipitation measurement was obtained at a median distance of their home of 16 and 19 km respectively.

Many measures of absolute humidity are used in the literature but we used the vapor pressure, which can be calculated from the mean air and dew point temperature to be consistent with recent findings [14]. First we calculated the relative humidity (RH) for moist air (assumption: RH
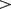
50%) from the dew point depression [39]:
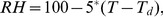
(1)with 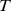 the air temperature and 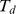 the dew point temperature. The average RH in Belgium throughout the year published by the Royal Meteorological Institute of Belgium (http://www.meteo.be/) is at least 72%, which validates the moist air assumption. Next, the saturation vapor pressure was derived from the temperature using the Clausius-Clapeyron relation [39]:
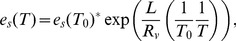
(2)with 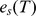 the saturation vapor pressure at temperature 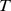, 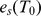 the saturation vapor pressure at the reference temperature, 6.11 mbar, 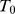 the reference temperature, 273.13 K, 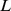 the latent heat of evaporation for water, 2260 kJ/kg and 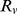 the gas constant for water vapor, 461.5 J/(kg*K). Finally, the vapor pressure or absolute humidity was calculated by:
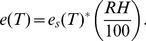
(3)


### Diary weights

The diaries were divided in sample populations according to the weather and/or the day it was filled in. We adopted the median of all daily temperatures and precipitation levels as thresholds to distinguish between days with high and low temperatures and between days with high and low precipitation. Second, the contribution of each diary was weighted to account for different sampling probabilities in each subpopulation. Information about the participants' age and household size and the day of the week the contacts were collected was used to calculate these weights [40]. Census data from 2001 for Belgium published by Eurostat (http://epp.eurostat.ec.europa.eu) was used as a reference and weights were constrained to a maximum of 3 to limit the influence of single participants.

### Mean number of contacts

We multiplied the total number of contacts from each participant with its diary weight and calculated the mean. We repeated this for contacts of specific types (home, work, school, other) and durations (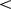
15 min, 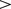
15 min, 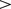
1 h, 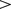
4 h). We furthermore looked at contacts involving skin-to-skin touching. Next, we measured relative changes in the number of contacts by dividing the weighted means from two conditions.

### R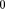 ratio

The contact data were used to calculate transmission rates for the sample populations [21], [32], [33]. The elements of the social contact matrix 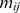, representing the mean number of contacts in age class *j* during one day reported by a respondent in age class *i*, can be estimated by the following expression:
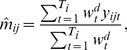
(4)where 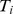 is the number of participants in age class *i*, 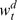 the diary weight for participant 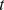 and 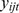 the reported number of contacts made by participant 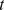 of age class 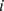 with someone of age class 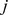. We used 7 age classes based in part on the Belgian schooling system to reduce sparse data cells in the contact matrix: 0–5 years, 6–12 years, 13–18 years, 19–25 years, 26–45 years, 46–65 years and over 65 years of age. For each sample population we calculated the mean number of contacts between all age groups using all contacts, contacts involving skin-to-skin touching or specific contact types and durations. The reciprocal nature of contacts requires 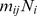 to equal 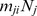, so we defined the elements of the social contact matrix 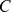, representing the per capita daily contact rate between age classes, as:
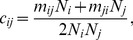
(5)with 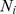 the population size in age class 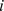, obtained from demographic data (Eurostat, 2010). This reciprocal behavior is not assumed for the specific contact types because for instance contacts for a salesman are most likely not contacts at work for the customers. Therefore, we defined the elements of 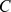 for these specific contact types as 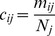.

The next generation matrix 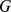 with elements 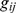 indicates the average number of secondary infections in age class 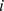 through the introduction of a single infectious individual of age class 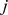 into a fully susceptible population [41]. The next generation matrix is defined by:
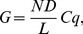
(6)with 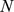 the population size, 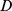 the mean duration of infectiousness, 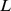 the life expectancy, 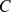 the contact matrix and 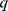 the proportionality factor. The basic reproduction number R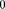 can be calculated as the dominant eigenvalue of the next generation matrix. To estimate the relative change in R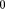 , we used the R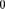 ratio:
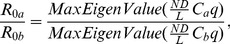
(7)where indices 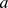 and 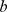 refer to the contacts registered during different weather conditions. The R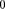 ratio can be estimated using only social contact rates when assuming 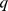 to be constant since the normalizing constants cancel. Albeit that this 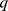 might vary by weather condition, assuming a constant 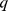 allows us to focus on the effect of modified social contact behavior.

### Bootstrapping

We used a nonparametric bootstrap on the social contact data by resampling participants to calculate 95% confidence intervals. Stratification by participant age was used in the bootstrap to maintain the original age distribution. To estimate the required number of bootstraps to obtain stable results, we calculated R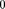 ratio confidence intervals with different bootstrap sizes.

## Supporting Information

Figure S1 **Age distribution of the sample populations after partitioning for day-type and daily temperature.** Top: regular workdays with low (less or equal than the median temperature, A1) and high (A2) temperatures, regular weekend days with low (B1) and high (B2) temperatures. Bottom: workdays during official holiday periods with low (C1) and high (C2) temperatures, weekend days during official holiday periods with low (D1) and high (D2) temperatures.
(TIFF)

Figure S2
**R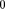 ratio confidence interval limits for different bootstrap sizes.** The upper and lower limits of the 95% confidence intervals (CI) for the ratio of the estimated R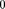 's for regular workdays with high and low precipitation.(TIFF)
