# Supplementary figures and images for: A Nice Day for an Infection? Weather Conditions and Social Contact Patterns Relevant to Influenza Transmission
Source: PLoS One. 2012 Nov 14;7(11):e48695. doi: 10.1371/journal.pone.0048695 (PMC3498265; doi:10.1371/journal.pone.0048695)

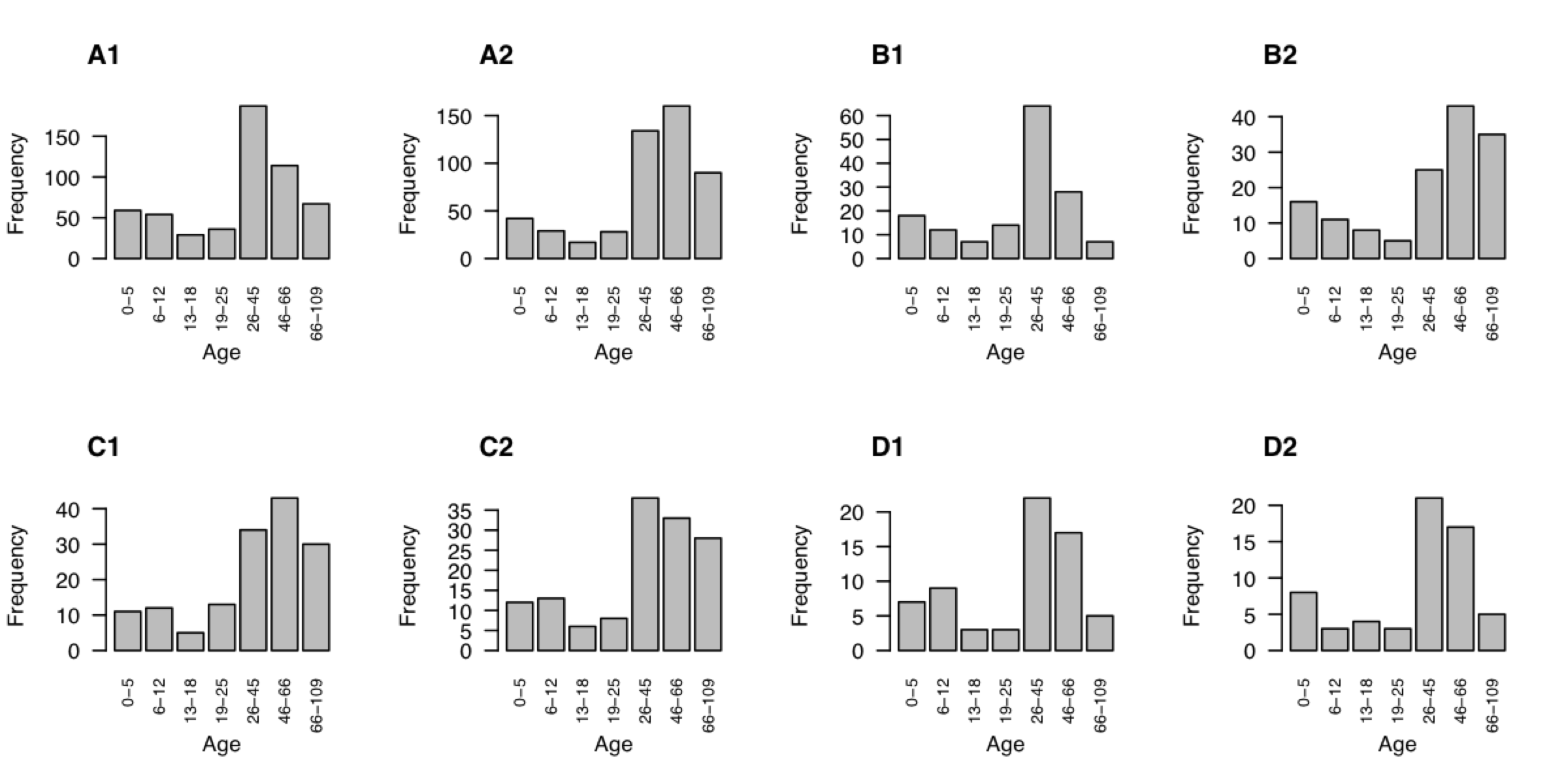

Supplement: Figure S1 — Age distribution of the sample populations after partitioning for day-type and daily temperature. Top: regular workdays with low (less or equal than the median temperature, A1) and high (A2) temperatures, regular weekend days with low (B1) and high (B2) temperatures. Bottom: workdays during official holiday periods with low (C1) and high (C2) temperatures, weekend days during official holiday periods with low (D1) and high (D2) temperatures. (TIFF) [file pone.0048695.s001.tiff]

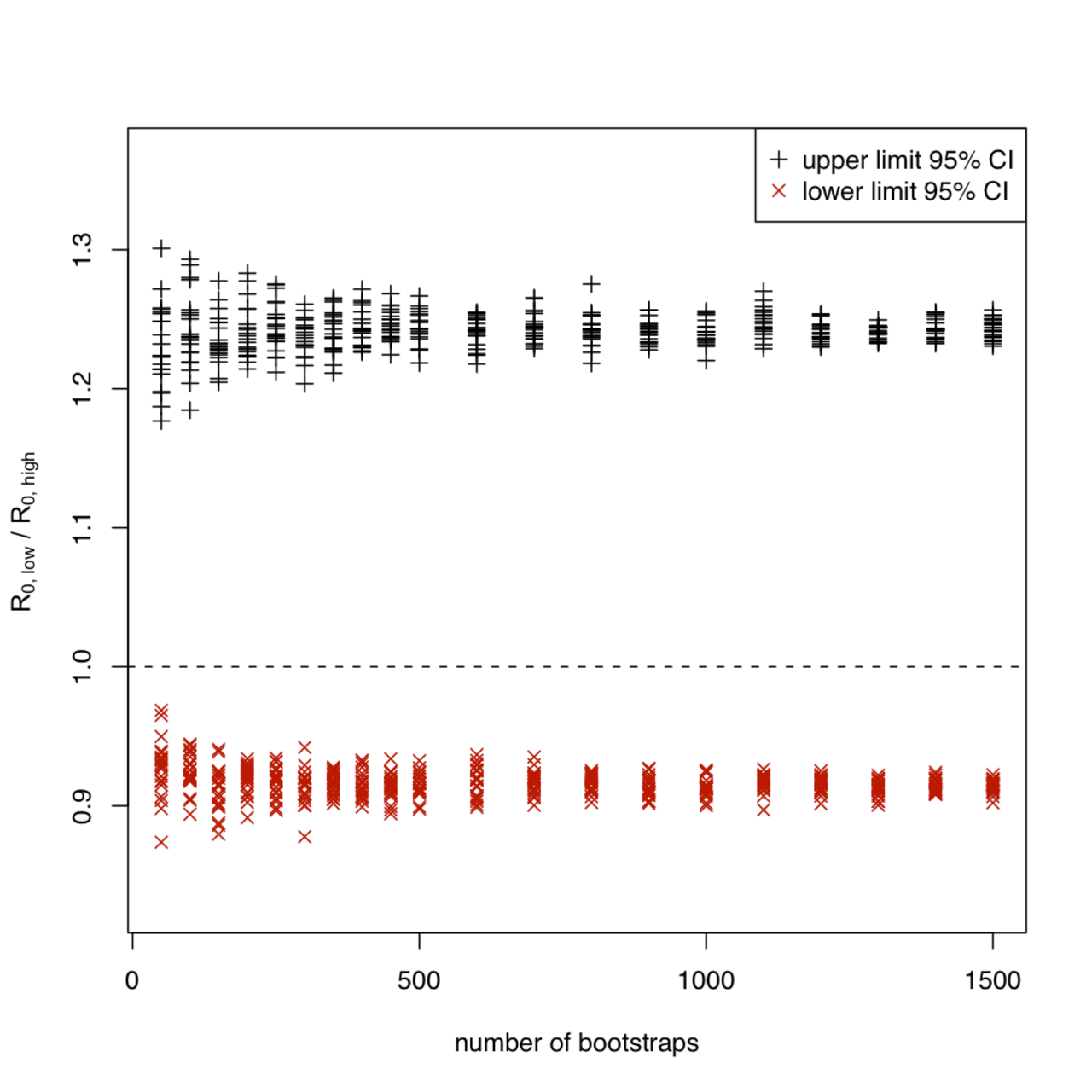

Supplement: Figure S2 — R ratio confidence interval limits for different bootstrap sizes. The upper and lower limits of the 95% confidence intervals (CI) for the ratio of the estimated R 's for regular workdays with high and low precipitation. (TIFF) [file pone.0048695.s002.tiff]
